# Successful removal of a stuck plastic stent in an echoendoscope by additional insertion of a transnasal endoscope

**DOI:** 10.1055/a-2387-3765

**Published:** 2024-09-10

**Authors:** Daisuke Namima, Toshio Fujisawa, Sho Takahashi, Yusuke Takasaki, Ko Tomishima, Shigeto Ishii, Hiroyuki Isayama

**Affiliations:** 1 73362 Department of Gastroenterology, Juntendo University School of Medicine Graduate School of Medicine, Bunkyo-ku, Japan

Various innovations have been reported as troubleshooting measures in response to the increasing adoption of interventional endoscopic ultrasound (EUS) 1 2 3 . We report herein a case in which a plastic stent, stuck in the working channel outlet of an echoendoscope (EG-740UT; Fujifilm, Tokyo, Japan), was successfully removed without echoendoscope withdrawal, and the guidewire was kept in place by additional insertion of a transnasal slim endoscope.

A 68-year-old man underwent EUS-guided hepaticogastrostomy (EUS-HGS) for intrahepatic bile duct stones secondary to hepaticojejunostomy stenosis (HJSS) ( Fig. 1 ). Stent exchange and additional stent insertion were performed to dilate the EUS-HGS tract and HJSS 1 month after the initial EUS-HGS. After placement of a guidewire in the bile duct beside the previously placed plastic stent using an echoendoscope, we attempted to remove the plastic stent with grasping forceps. However, the plastic stent had become stuck in two folds in the working channel outlet of the echoendoscope ( Fig. 2 ). We tried unsuccessfully to push and pull the plastic stent with the grasping forceps. Because passing the liver parenchyma had been difficult in the initial EUS-HGS, we kept the guidewire in place because reinserting it would be difficult if it were once withdrawn. We attempted to retrieve the stuck plastic stent by additionally inserting a slim transnasal endoscope (GIF-XP290N; Olympus, Tokyo, Japan) while maintaining the orally inserted echoendoscope. First, the portion of the plastic stent protruding outside the echoendoscope was grasped with a small-caliber snare ( Fig. 3 ). Next, the plastic stent was successfully removed by pulling out the slim transnasal endoscope. The guidewire was kept in the bile duct, and subsequent procedures were smoothly performed. Finally, we successfully placed two plastic stents in the EUS-HGS tract, bridging the HJSS ( Video 1 ).

**Fig. 1:**
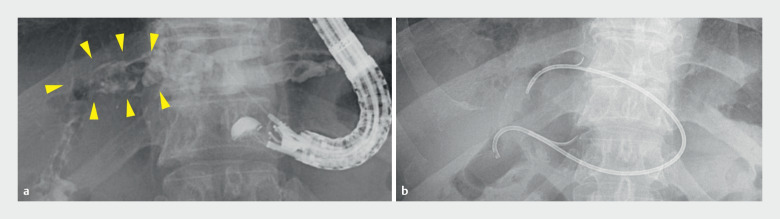
Initial endoscopic ultrasound-guided biliary duct drainage in a 68-year-old man. **a** Contrast injection revealed intrahepatic bile duct stones (arrowheads) secondary to hepaticojejunostomy stenosis. **b** A 7-Fr plastic stent was placed to connect the jejunum, bile duct, and stomach.

**Fig. 2:**
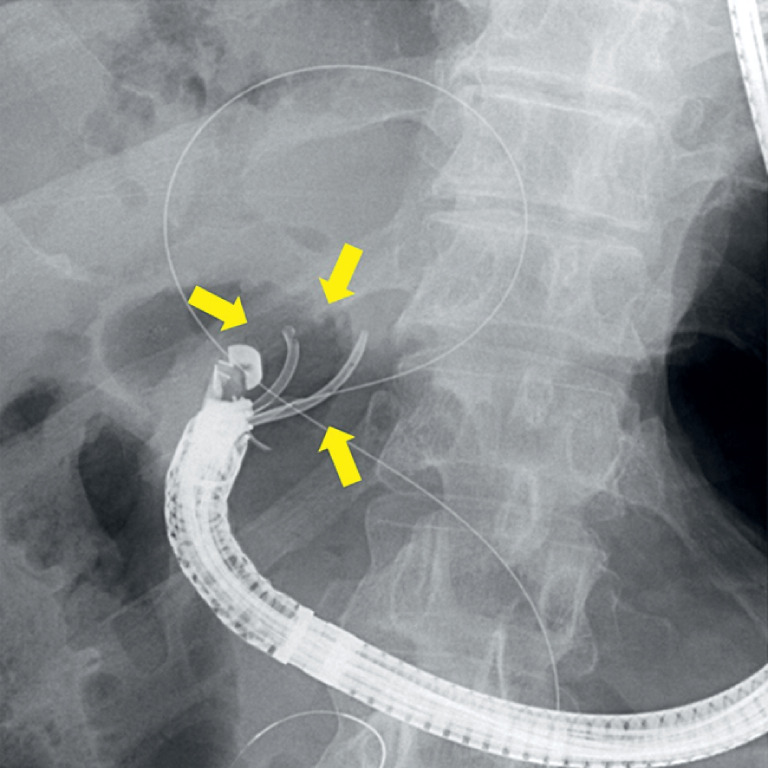
During stent exchange and additional stent insertion 1 month later, to dilate the EUS-HGS tract and HJSS, the previously placed plastic stent became stuck in two folds in the working channel outlet of the echoendoscope (arrow).

**Fig. 3:**
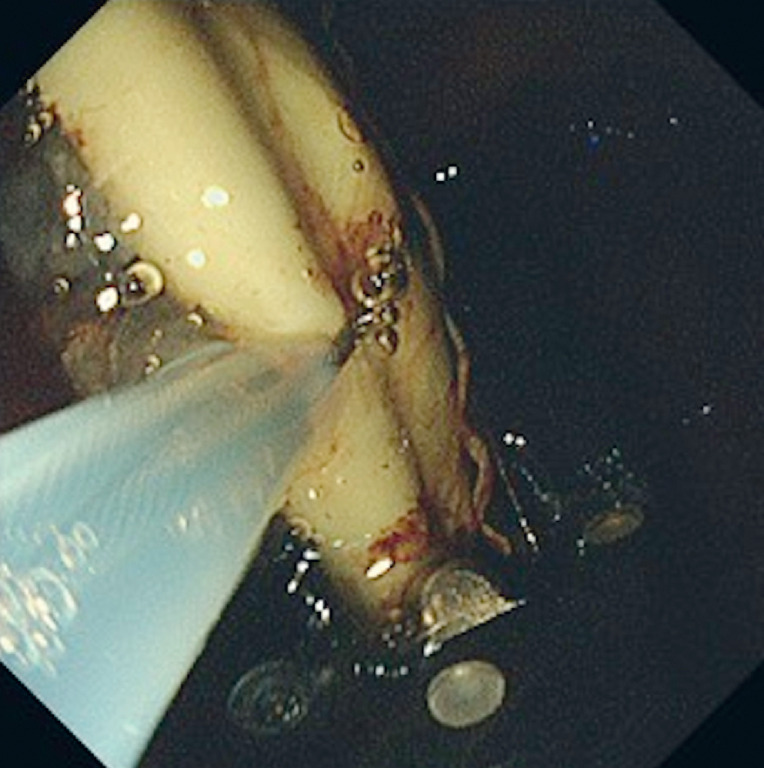
The portion of the plastic stent protruding outside the echoendoscope was grasped with a small-caliber snare.

Successful removal of a stuck plastic stent in an echoendoscope by additional insertion of a transnasal endoscope. GW, guidewire; PS, plastic stent.Video 1

The simultaneous use of two endoscopes has been reported in various endoscopic procedures and is considered a valuable troubleshooting option 4 5 .

Endoscopy_UCTN_Code_CPL_1AL_2AD

## Notes

Endoscopy E-Videos https://eref.thieme.de/e-videos
*E-Videos* is an open access online section of the journal *Endoscopy* , reporting on interesting cases and new techniques in gastroenterological endoscopy. All papers include a high-quality video and are published with a Creative Commons CC-BY license. Endoscopy E-Videos qualify for HINARI discounts and waivers and eligibility is automatically checked during the submission process. We grant 100% waivers to articles whose corresponding authors are based in Group A countries and 50% waivers to those who are based in Group B countries as classified by Research4Life (see: https://www.research4life.org/access/eligibility/ ). This section has its own submission website at https://mc.manuscriptcentral.com/e-videos .
